# Quarterly Retrospect

**Published:** 1862-10

**Authors:** 


### *Quarterly Retrospect.*

*October*, 1862.

In the celebrated discussion upon the nose of Slawkenbergius, the learned, it will be remembered, strove to arrive at Truth by pumping her up through the conduits of dialectic induction. They concerned themselves not with facts—they reasoned. By a somewhat similar process the dogma of the sufficiency of common observation in determining the existence of, or for rightly dealing with insanity in legal cases was, we presume arrived at by its distinguished propounders. The facts happen otherwise, we assert,. and the two following cases may be cited in support of the assertion from the judicial records of the quarter.

John Roberts was tried before Mr. Justice Blackburn, at Gloucester, for the murder of Clara Roberts, a child about sixteen months old. He had beaten the child about the head with a poker whilst she was lying in bed with a sister, about five years of age. When arrested, the prisoner remarked to the policeman, "Very well; I'll go quietly with you. It was owing to drink and bad temper." In defence of Roberts, it was asserted that he was of unsound mind at the time of the murder, and in support of this assertion it was sworn in evidence that the prisoner would sometimes come down stairs with his clothes upon his arms, and attempt to go out into the streets in a state of nudity; that when a boy he used to preach in the streets, and the boys made sport of him; that his grandfather had been insane for twelve months before his death, and that an uncle had also been insane and confined for two years in an asylum at Stoke Bishop. No medical evidence was adduced in support of the averment of insanity, and in summing up, Mr. Justice Blackburn observed that "the evidence of insanity in this case was but slight, and it was much to be regretted that Dr. Mayor and other medical men who had been referred to, were not present. Their absence was much to be regretted, but that was probably owing to the poverty of the prisoner.

The jury returned a verdict of "*Not Guilty* on the ground of insanity."

Pursuing the customary routine in such cases, the Secretary of State called upon the visiting justices to send him a certificate of the prisoner's insanity, in order that he might be removed to a lunatic asylum. He was consequently examined by the gaol surgeon, Dr.

Bleeck, and by Dr. Lyon, but neither gentleman could discover any symptom of lunacy. The magistrates, therefore, have been under the necessity of reporting that so far as can be ascertained, the prisoner is in full possession of his senses.

In the recent celebrated case of George Clark, the jury gave a verdict of Guilty, the prisoner at the bar being at the time incoherent as well as suffering from delusions; in the present case the converse has occurred, and without any but the very slightest evidence of mental disorder the jury acquit the prisoner on the ground of insanity.

An action (Scott *v.* Wakem) was lately tried before Mr. Baron Bramwell, in which the plaintiff complained that the defendant, a medical man, and others, had entered the plaintiff's dwelling-house, and assaulted and beat him, and caused him to be imprisoned and kept under personal restraint.

The plaintiff was a retired naval officer, residing on his means, at Kennington. The defendant was a medical man living in the neighbourhood, who had attended the plaintiff for a few weeks before the day in question. On that day, the 10th of February, according to the case for the plaintiff, he had been drinking, and as there were loaded pistols of his in the house, the wife sent the servant to the police station for a policemen to unload the pistols for fear of danger; and the defendant, hearing of this, came to the house at one o'clock, and again about six, to see the plaintiff; and then, without any necessity or authority, sent a man named Streets, who kept the plaintiff under restraint all night. The plaintiff and his wife, while admitting that he had been drinking in the early part of the day, stated that he had had a sleep, and was then sober, or at least so far recovered that there was not any danger; and the wife stated that she told the defendent in the evening, when he proposed to send some one to take charge of her husband, that there was then nothing the matter with him, and that the defendent need not send any one. And the case for the plaintiff was supported not only by himself and his wife, but by the servants and two other witnesses.

The defendant when called, said he was surgeon to the Queen's Prison and to a division of police. He had attended the plaintiff from the 19th of December to the 22nd of January, previous to his visit to Paris. He stated that the plaintiff had been subject to *delirium tremens*, and had once come into his surgery and threatened him with loaded pistols. On the day in question he was sent for from the plaintiff's by a woman, who rushed in, and said, "For God's sake, come! he is going to murder his wife." He went, and found the plaintiff in a most violent and excited state, with loaded pistols in his hands, threatening to shoot his wife, and two men holding him He was, the witness said, in a fit of *delirium tremens*, and in a very hangerous state. He said the wife asked him to sign a certificate for his removal from the house, but he declined doing so, as, if a strait-waistcoat were put upon him in such a state, he might probably die of excitement. The wife also asked him to send some one to take care of the plaintiff, and he sent Streets, an invalid nurse, to do so. At ten o'clock at night he went again, and found the plaintiff much better; and, though still incoherent in his language, not so violent. The wife asked that Streets should not go away, lest the plaintiff should take to drinking again, and Streets remained at her request. Next morning the defendant found the plaintiff better, though still trembling, and he asked defendant to explain to him what had occurred. The defendant said he told him what had taken place, and the plaintiff thanked him for what he had done.

Streets, who described himself as an attendant on insane or nervous gentlemen, and said he had been employed in that capacity for many years by medical men, and had had charge of many cases of *delirium tremens*, said he had been employed on this occasion by the defendant, whom he had known for some time. He had known patients in *delirium tremens* wake up suddenly from sleep and attack people. He went upstairs to see the plaintiff at the request of the women who were at his residence. He thought Mrs. Scott, the plaintiff's wife, was one of them. He found the plaintiff standing in the middle of the room undressed—with nothing on but his trousers—washing himself. The plaintiff asked him who he was, and the witness said it did not matter, but he was sent for because he had heard that the plaintiff had been violent, and he proposed that they should "talk things over quietly." The plaintiff then suggested supper and sherry, and the witness said, "Anything in moderation." A bottle of sherry was sent for, and a third of it sent up to the plaintiff with his supper. Witness left him, and went downstairs, and while he was away the plaintiff got the rest of the wine, and emptied the bottle. The witness described the plaintiff's conversation with him, which, according to his account, was senseless and irrational. He said that when the plaintiff went to bed the wife asked him to stay in the house, and he remained downstairs all night, she being with her husband. There was no further disturbance during the night, and all was quiet. Next day the witness saw the plaintiff, who thanked him for his kindness, and said that the defendant had asked him to give him ten shillings, but he should give him a sovereign. The witness stated that once a patient under *delirium tremens* suddenly woke up from sleep, took the poker, and very nearly knocked him on the head.

In answer to the learned JUDGE,

The witness said that when he first saw the plaintiff he was quietly washing himself, and he could not say he was excited, but he seemed a little excited when he saw a stranger.

The Jury returned a verdict for the plaintiff: Damages one farthing.

The utter absurdity of this verdict was pointed out with singular aptitude in the following admirable and pungent letter which appeared in the *Times* (August 14th), addressed to the editor:—

SIR,—Allow me, as a member of the medical profession, to return my humble acknowledgments to the jury who tried the cause of "Scott *v.* Wakem," as reported in your impression of to-day, for the following important lessons which we may derive from their verdict:—

"1. That in any case of delirium or mania it is unsafe for a medical man to attend unless summoned expressly by the patient himself.

"2. That, when attending, it is unsafe for him to employ any means of restraint without first obtaining the consent of the patient himself.

"3. That on neither of these points is the authority of the patient's wife or relatives sufficient to guard the medical attendant from legal consequences.

"4. That between insanity and *delirium tremens* a broad line of demarcation must be drawn, a person in the latter condition being incapable of injuring either himself or others.

"5. That the fact of a patient 'standing in the middle of his room, undressed, and washing himself,' is proof positive of his possessing a *mens sana in corpore sano*, and of his being far removed from the necessity of personal restraint."

Deeply impressed, Sir, with a sense of the important services they have thus rendered our profession, and with their unquestionable fitness for such a task, I would humbly request these twelve enlightened men to lay down a code of rules for our guidance when summoned by terrified relatives to the treatment of a raging maniac as may perchance, if followed, save us from the annoyance and loss of time consequent upon action at law, to say nothing of being mulcted in one farthing damages with costs. And, as a last favour, I would entreat these gentlemen to append their names to such rules, both to give force and authority to them, and also to enable us in our gratitude to hand down to an admiring posterity the cognomens of our benefactors. To grant these requests would surely be no more than simple justice to the members of the medical profession, who in dealing with such cases of mania are morally, if not legally, responsible for the limbs and lives of their fellow-beings; and themselves frequently incur great danger and sometimes, as in a recent case, even death, and whose position, as at present defined, appears to be somewhat as follows:—That if when called to cases of this description they refuse to obey the summons, they are liable to punishment for neglecting their duty; that if, being in attendance, they should proceed to use such restraint as in the exercises of their professional skill and judgment they deem necessary to insure the safety of the patient and attendants, they are liable to punishment for exceeding their duty; that if, deterred by dread visions of future legal proceedings looming in the distance, they hesitate to adopt the necessary restraint, and any casualty should consequently occur, then again they are liable to punishment for neglecting their duty.

I am, Sir, your obedient servant,

A PUZZLED PRACTITIONER.

The question of eccentricity has once more arisen in the course of a trial for murder. A Newcastle jury found the lunatic Clark guilty of murder, and Mr. Justice Willes, in sentencing him to death, said that he was convinced that the prisoner was a person of "eccentric conduct and violent character," and, again, that his "character was eccentric beyond that of other men;" but not a word of mercy came from either judge or jury. An Irish jury have set a somewhat different example.

Wm. Herdman was tried at Belfast, before the Right Hon. Justice Fitzgerald, for the murder of Mr. John Herdman. The facts stated in evidence of insanity, were as follows:—

Mr. John Atkinson, who believed he was a relation of the prisoner, knew him upwards of thirty years ago. He had extraordinary eccentricities in his youth. Witness knew him to blow horns in the streets, and to go up and down Sackville-street, Dublin, with a false nose and a mask on, carrying the handle of a sweeping brush as a walking-stick; he would tear five or six leaves out of a book, and take them to read in bed, to avoid carrying the book; he frequently said he felt a sudden desire to kill the witness—"to knock his fist through him." He was in the habit of carrying fire-arms, and had worn a coat of mail under his dress. He had often said that he would spend all his money, and then blow his brains out. He would hire a car, get into the driver's seat, drive round the city, and get arrested by the police. Mr. Charles Eccles, a magistrate, knew the prisoner twenty-three years ago, when he was impulsive and eccentric, delighting to beat trays and drums, shouting and yelling, and (meaning the noise) would exclaim, "This is Heaven!" At church he behaved very well till the singing commenced, when he burst out laughing, and ran out of church. (This might mean only that he had an exquisite ear for music, which was tortured beyond endurance.) Charles Macartney, formerly a first-class constable, now a pensioner, deposed that in 1848 the prisoner issued a placard with his name attached, summoning a Repeal meeting at Ballyshannon, which, however, he did not venture to address. Mr. John Thompson, proprietor of the *Armagh Guardian*, had the prisoner once in his employment, and thought him singular; he had a peculiar eye, was wild-looking, and he set him down as insane. Robert G. Herdman, the prisoner's son, fifteen years of age, deposed that his father carried pistols, generally loaded; that he seemed at times very absent, lost in reverie, and would not seem to hear when spoken to; that he carried bottles containing essential oil of bitter almonds suspended from his neck with strings; that he would often put his hat on a hedge, and walk bareheaded; that he would get up at three in the morning if he heard a dog bark or a cock crow, and call on a policeman to have them removed; that he would take lodgings and never go to them, &c.

Surgeon Wells stated that he had been a fellow-passenger with the prisoner to America, in 1854, when he exhibited symptoms of an unsound mind, and was dangerous. He considered him subject to paroxysmal insanity.

Mr. Richard Lilburn, editor of the *Armagh Guardian*, knew the prisoner in his office about five years ago. He was continually taunting the witness for being too tame in whatever he wrote; he said he would have the command of a newspaper, and he would literally "rend the world to blazes." He would laugh and cry without apparent cause. He talked of having spent large sums on bridles and spurs. He was continually boring him with doggrel poetry.

Mrs. Belinda Herdman, sister-in-law of the late Mr. Herdman, deposed that the prisoner was a cousin of the late Mr. Herdman. From repute in the family, she understood that his father hanged himself, and it was understood that a sister of the prisoner had drowned herself. He had another sister who was rather silly. His mother was very eccentric in dress; she wore eight or ten caps at the same time, and two bonnets when she went out. She had her bed covered with rags, and everything that would keep out the air.

Dr. T. H. Purdon, who had thirty-five years' experience of insanity, deposed that having heard the evidence, he believed the prisoner was of unsound mind. The witness was cross-examined at great length on the nature of insanity, and the alleged taint of madness in the prisoner's family.

The Crown then entered on a rebutting case, and produced Dr. Samuel Browne, who, after a practice of twenty years in Belfast, was decidedly of opinion that the prisoner was sane.

Mr. Hamill reviewed the evidence for the defence, and the Attorney-General replied at great length.

In summing up, his Lordship observed of one part of the evidence of the prisoner's son:—"The fact of his father carrying pistols about—loaded pistols—without having any necessity for them, but more particularly the fact of his keeping those bottles of poison hung about his neck, were more than eccentricities."

The jury returned a verdict of Guilty, adding that they did not think the prisoner insane in the strict sense of the word, but they thought his eccentricities were quite sufficient to warrant their unanimous recommendation to mercy.

The prisoner manifested little emotion. The sentence has been commuted to penal servitude for life.

